# The Hahnemann Monument

**Published:** 1896-03

**Authors:** J. H. McClelland

**Affiliations:** Chairman Monument Committee, Am. Inst. of Homœopathy


### THE HAHNEMANN MONUMENT.

TO THE EDITOR OF THE HOMŒOPATHIC PHYSICIAN.

DEAR DOCTOR:—Hahnemann's birthday is approaching and should be made the occasion of a great demonstration. We ourselves must honor Hahnemann if we would have him honored. This year also marks a great Centennial. It is just one hundred years since Hahnemann published in *Huffland's Journal* his famous paper on "A New Principle for Ascertaining the Curative Powers of Drugs." This was the first gun fired in the mighty revolution in medicine which has transpired in this century.

It has been proposed by Dr. Bushrod James that Hahnemann's birthday should be made the occasion of a contribution by every homœopathic physician in the United States to the monument. The models of the monument are about completed and it will be a magnificent memorial. No homœopathic physician can afford to have his name missing in the list of contributors.

Let the contributions be sent at once to Dr. Henry M. Smith, 288 St. Nicholas Avenue, New York, and they will be promptly acknowledged. A list of contributors is soon to be published and should include every member of the profession. A grand rally and the work is done.

Fraternally yours,

J. H. McClelland,

*Chairman Monument Committee, Am. Inst. of Homœopathy.*

